# Novel Targets and Mechanisms in Antimicrobial Drug Discovery

**DOI:** 10.3390/antibiotics10020141

**Published:** 2021-02-01

**Authors:** Donatella Tondi

**Affiliations:** Department of Life Sciences, University of Modena and Reggio Emilia, Modena 41125, Italy; donatella.tondi@unimore.it

The spread of infections resistant to available anti-infective drugs is a serious menace to human health. WHO predicts that in less than 30 years, microbial resistance will become the leading cause of death. However, the pipeline of new anti-infective drugs close to being approved for therapy, or those that are presently under development, is scarce.

To fight resistant microorganisms, both the discovery and validation of new targets are highly desirable, and only innovative drugs with new modes of actions that are able to escape preexisting mechanisms of resistance could represent a valid solution to counteract the continuing emergence and spread of resistant infections.

This Special Issue comprises a total of 11 contributions of full research articles, short communications, and reviews, providing a glimpse into the state of the art of medicinal chemistry research devoted to microbial resistance.

Among the proposed subjects herein, bacterial resistance to available antibiotics is largely represented, with the scientific community driven in finding rapidly new antibiotics to overcome resistance in bacteria.

The possibility offered by biofilms to hit drug-resistant infections is the focus of the contribution by Seyler et al. [1]. Biofilm indeed represents an innovative target for the control of resistant strains. The authors reveal new anti-infective molecule-inhibiting biofilm growth by targeting the tRNA-dependent regulated T-box genes modulating the expression of aminoacyl-tRNA synthetases and amino acid metabolism genes. Active molecules were identified via in silico screening and validated in vivo, whereby the growth of *Staphylococcus aureus* in biofilms was inhibited 10-fold more potently than by vancomycin. Moreover, for the identified compounds, synergy was detected when administered in combination with gentamicin and rifampin. The selected target and the results obtained highlight the importance of hitting critical and specific bacterial functions that are absent in human hosts but required for bacterial cell viability. T-box is a unique target that could be exploited in the development of small-molecule antibacterial biofilm therapies against virulent and drug-resistant Gram-positive pathogens.

In their contribution, Hennessen and coworkers discuss the ability of amidochelocardin, a biosynthetic derivative of the bioactive NP chelocardin, to overcome known bacterial resistance mechanisms [2]. Amidochelocardin acts as a broad-spectrum antibacterial that is active against the ESKAPE group of clinically relevant bacteria. The ability of this atypical tetracycline to escape common resistance mechanisms, i.e., efflux processes, was investigated and validated against a large panel of multidrug-resistant (MDR) uropathogenic clinical isolates. The molecule represents a promising candidate to be developed into future therapeutics.

Antimicrobial resistance among *S. aureus* was investigated by Kavaliauskas and coworkers [3]. The authors discuss a series of 5-nitro-2-thiophenecarbaldehyde derivatives. As such, for the most active molecule, the in silico structure-based pharmacological properties and toxicity were predicted. In biological assays, the compound significantly impaired the *S. aureus* biofilm integrity, demonstrating good antibacterial activity against multidrug-resistant *S. aureus*. The results obtained suggest the therapeutic potential of the identified lead as a vancomycin-resistant *S. aureus* (VRSA)-targeting antimicrobial agent.

The research article by Reidl et al. deals with *N*-acetyl-6-sulfonamide indoline derivatives as inhibitors of DapE [4]. This bacterial enzyme, which is indispensable for bacterial survival and pathogenesis but absent in humans, has been recently validated as a promising bacterial target for the design of antibiotics with a new mechanism of action. For a series of prepared derivatives, molecular docking predictions suggested a mechanism of action based on a specific interaction within the DapE ZN (II) binding site. This study can represent a starting point for the drug design of small, drug-like DapE inhibitors as potential, highly selective antibiotics.

Fiore et al. contribute to the issue [5] with a systematic evaluation of the effectiveness of the ceftazidime–avibactam (CZA) combination, which was recently approved for the treatment of severe infections. The authors performed a systematic review and network meta-analysis (NMA) with the aim of comparing the effectiveness of CZA monotherapy versus combination therapy with other antibiotics in terms of mortality in patients with carbapenemase-producing Enterobacteriaceae (CRE) infections.

The authors included both randomized controlled trials (RCTs) and nonrandomized studies as per MEDLINE by PubMed, EMBASE, and the Cochrane Central Register of Controlled Trials. In total, 13 studies were finally included in the qualitative synthesis and six studies in the final meta-analysis. The main findings of this study indicated that there were no significant differences in mortality in the treatment of CRE infections with CZA combination therapy compared to CZA monotherapy. This evidence may be useful for optimizing antibiotic treatments, with the potential to reduce the use of combination treatments.

Moving to the design of agents active in tuberculosis (TB) infection, Venugopala et al. explore the functionalization of the triazole nucleus and its impact on anti-TB activity [6]. Selected derivatives were validated against H37Rv and MDR strains of *Mycobacterium tuberculosis* (MTB). Molecular docking and molecular dynamic (MD) simulation conducted for the most promising compounds identified β-ketoacyl ACP synthase I (KasA) as the potential cellular target of the detected anti-TB activity. The results can support further hit-to-lead development of triazolyl anti-TB agents with improved potency and selectivity.

Besides their contribution to the anticapsular and antifungal activity of α-cyperone, Horn et al. focus their attention on novel drugs to fight virulence and resistance in fungal pathogens [7]. The authors screened a collection of medicinal plant sources looking for antifungal activity, thus identifying an active fraction from the rhizome of *Cyperus rotundus*, the nut grass plant. The essential oil α-Cyperone demonstrated fungicidal activity against different species of *Candida*. Moreover, when combined with a clinical antifungal drug, fluconazole, the minimal inhibitory concentration of α-Cyperone was reduced. Further work needs to be carried out at the mechanistic level to fully validate α-Cyperone as a novel, synergistic antifungal agent. 

The work by Ngnameko et al., likewise covers the possibility offered by natural sources for the identification of bioactive molecules by reporting on the medicinal properties of *Spathodea campanulata* P. Beauv. (Bignoniaceae) [8]. The work shows preliminary evidence on the ability of the crude plant extract to inhibit *Helicobacter pylori* growth by urease inhibition and modulation of virulence factors. Fractions and subfractions of the plant crude extract were characterized by ultrahigh-performance liquid chromatography and high-resolution mass spectrometry (UHPLC–HRMS). Kaempferol, a flavonol, was identified as a promising compound. Further investigations are ongoing to confirm the capacity of this new hit to modulate *H. pylori* virulence factors.

Among the contributed reviews, Panchal et al. propose a complete review of riboswitches (RB) and lead compounds that have been identified so far against various riboswitches via fragment screening, high-throughput screening, and structure-based design [9]. The authors discuss the current status of research in the field and the potential offered by RB. Their ability to regulate genes, specifically those involved in the biosynthesis of essential metabolites in pathogenic bacteria, de facto render them a potent and druggable antibacterial target for the development of antibiotics with a new mechanism of action. The authors highlight challenges in the identification of novel active compounds, in particular the lack of specific high-throughput screening methods and the risk of hitting off-targets with nonselective molecules.

Gianquinto and coworkers [10] propose a review of β-lactamase (BL), the bacterial enzymes representing a major resistance mechanism to β-lactam antibiotics in Gram-negative bacteria. The interest in discovering novel BL inhibitors is high as the more newly emerged BLs are not inhibited by the available inhibitors. Moreover, the insurgence rate of effective mutations is far higher than the discovery of new inhibitors able to counteract them, slowing down the approval of new BL inhibitors in therapy. In relation to this, the authors discuss a strategy to target allosteric sites in BLs, thus overcoming the frequent occurrence of mutations and the difficulty of competing efficaciously with substrates. Allosteric inhibitors could work synergistically with traditional inhibitors, increasing the chances of restoring bacterial susceptibility toward available antibiotics.

Cryptic and druggable sites recently found on BLs, as well as identified allosteric effectors are discussed by the authors. However, challenging questions remain open, i.e., the lack of consensus on the chemical features a ligand should have for behaving as an allosteric inhibitor and the difficulty in impairing cross-class activity because of the different structures and folding characteristics among different BLs.

Impey et al., close the Special Issue with a review dedicated to bacterial targets within the most well-characterized resistance mechanisms associated with the cell wall of Gram-negative bacteria, such as the outer membrane structure, porins, and efflux pumps. A timely update on the current progress of inhibitor development in these areas is also provided [11].

This Special Issue collects multidisciplinary contributions focused on the development of anti-infectives with a special glimpse at novel, promising targets. The collected contributions constitute a valuable knowledge reservoir for scientists working in the field of drug discovery devoted to overcoming antimicrobial resistance.
